# Supplementary material for: ProteinShader: illustrative rendering of macromolecules
Source: BMC Struct Biol. 2009 Mar 30;9:19. doi: 10.1186/1472-6807-9-19 (PMC2672931; doi:10.1186/1472-6807-9-19)
Supplement: Additional file 1 — ProteinShader program without source code. This compressed file contains the complete ProteinShader program including associated libraries, but no source code. A README.txt file gives an overview of the ProteinShader distribution, and the index.html file in the help subdirectory has directions on getting started with the program as well as a set of tutorials. [file 1472-6807-9-19-S1.zip › ProteinShader-beta-0_9_4-binary/help/api/org/proteinshader/structure/package-summary.html]

org.proteinshader.structure (ProteinShader API)


|  |  |  |
| --- | --- | --- |
| **Overview** \| **Package** \| Class \| **Use** \| **Tree** \| **Deprecated** \| **Index** \| **Help** | |  |
| **PREV PACKAGE**   **NEXT PACKAGE** | **FRAMES**    **NO FRAMES**     **All Classes** |  |


---

## Package org.proteinshader.structure

Holds the classes that store information from a Protein Data Bank file:
Structure, Model, Chain, AminoAcid, Heterogen, Water, Atom, Bond, Helix,
BetaStrand, Loop, *etc*.

**See:**
  
          **Description**

| **Interface Summary** | |
| --- | --- |
| **IDTest** | This interface declares a processID() method that is intended to be used as a helper method for constructors that need to perform tests and/or any modifications on an ID. |

| **Class Summary** | |
| --- | --- |
| **AminoAcid** | This concrete subclass of the abstract class Residue stores information on an amino acid in a PDB structure entry. |
| **Atom** | Extends abstract class Drawable to add data that is unique to an atom (atomID, type, temperature, occupancy, radius, *etc.*). |
| **BetaStrand** | This concrete subclass of Region holds information specific to a protein BetaStrand. |
| **Bond** | Extends abstract class Drawable to add data that is unique to a bond between two atoms. |
| **Category** | A Category object serves as a container for the Records in a PDB structure entry. |
| **Chain** | A Chain serves as a container for Residues (AminoAcid, Heterogen, and Water) and Regions (Loop, Helix, and BetaStrand). |
| **Description** | A Description object serves as a container for the Category objects that hold Records describing a PDB structure entry. |
| **Drawable** | This abstract class defines attributes and methods that are shared by all drawable objects, including the xyz-coordinates for the Drawable's center and methods that will allow each object to be sorted based on distance from the camera. |
| **Helix** | This concrete subclass of Region holds information specific to a protein Helix. |
| **Heterogen** | This concrete subclass of the abstract class Residue stores information on a heterogen in a PDB structure entry. |
| **Loop** | This concrete subclass of Region holds information on the general "loop" regions found between well-defined regions of secondary structure (helices and beta-strands). |
| **Model** | A Model serves as a container for one or more Chains. |
| **Record** | A Record object serves as a container for the lines of a PDB record. |
| **Region** | Abstract class that contains references to a region of a Chain of AminoAcids. |
| **Residue** | This abstract class serves as a container for one or more Atoms. |
| **Segment** | When a protein is drawn as a tube-like structure, a Segment is a length of the tube that corresponds to an AminoAcid. |
| **Structure** | Structure is the top-level container for objects that store information on a protein structure from the Protein Data Bank. |
| **Water** | This concrete subclass of the abstract class Residue stores information on a water molecule in a PDB structure entry. |

## Package org.proteinshader.structure Description

Holds the classes that store information from a Protein Data Bank file:
Structure, Model, Chain, AminoAcid, Heterogen, Water, Atom, Bond, Helix,
BetaStrand, Loop, *etc*.
